# Personalized radioiodine therapy for thyroid cancer patients with known disease

**DOI:** 10.12703/r/10-36

**Published:** 2021-04-07

**Authors:** Sissy M Jhiang, Peng Cheng, Fadi A Nabhan, Jennifer A Sipos, Chia-Hsiang Menq

**Affiliations:** 1 Department of Physiology and Cell Biology, The Ohio State University, Columbus, OH 43210, USA; 2 Department of Mechanical and Aerospace Engineering, The Ohio State University, Columbus, OH 43210, USA; 3 Division of Endocrinology and Metabolism, Department of Internal Medicine, The Ohio State University Comprehensive Cancer Center, Columbus, OH 43210, USA

**Keywords:** Personalized Radioiodine Therapy, Thyroid Cancer

## Abstract

Radioactive iodine (RAI) ^131^I is a targeted therapy for patients with RAI-avid follicular cell-derived thyroid cancer. However, the responsiveness to ^131^I therapy varies among thyroid cancer patients mainly owing to differential RAI uptake and RAI radiosensitivity among patients’ lesions. A personalized approach to maximize ^131^I therapeutic efficacy is proposed based on recent scientific advances and future opportunities.

## Introduction

Thyroid follicular cells have the ability to take up and retain iodide, a process that can be selectively enhanced by thyroid-stimulating hormone (TSH). This enables the use of radioactive iodine (RAI) for imaging and targeted killing of residual and/or metastatic RAI-avid thyroid cancer lesions following thyroidectomy. The gamma ray-emitting ^123^I and the positron-emitting ^124^I are used for diagnostic SPECT and PET imaging of RAI-avid lesions for therapeutic planning. The beta-particles emitted from ^131^I kill RAI-avid tumor cells and concomitantly emitted gamma rays allow imaging of RAI-avid lesions at therapeutic doses. While ^131^I has been a targeted therapy for well-differentiated follicular cell-derived thyroid cancer with a limited spectrum of adverse effects for almost 80 years, ongoing controversies remain in patient selection and optimal activity administration of ^131^I therapy^1^. As stated in the Martinique Principles^1^, the objective of ^131^I therapy can be categorized into remnant ablation, adjuvant treatment, or treatment of known disease. In this article, we will summarize the obstacles and opportunities in achieving personalized radioiodine therapy for thyroid cancer patients with known disease after thyroidectomy. Specifically, we will focus on strategies maximizing the therapeutic efficacy of ^131^I for individual patients.

## How do we increase ^131^I delivery to target lesions?

The ideal ^131^I treatment is to deliver sufficient ^131^I radioactivity to cure or stabilize progressive disease with minimal adverse effects. Thus, it is desired to increase ^131^I delivery to target lesions per administered ^131^I radioactivity. Established strategies to enhance ^131^I absorbed dose in target lesions include decreasing stable iodine intake, increasing TSH level via thyroxine withdrawal, or administering recombinant human TSH prior to RAI treatment. Patients who harbor RAI non-avid lesions on diagnostic scan under the aforementioned established strategies are less likely to be responsive to ^131^I therapy. Recently, MEK and/or BRAF^V600E^ inhibitors have been shown to redifferentiate RAI non-avid lesions into RAI-avid lesions, enabling some of these patients to benefit from ^131^I therapy with a structural response (see recent reviews 2,3). Patients carrying tumors with a *BRAF* or *RAS* mutation would be expected to demonstrate evidence of redifferentiation after taking a BRAF/MEK inhibitor; however, not all of them do. Very recently, Saqcena *et al.*^4^ showed that SWI/SNF chromatin remodeling complexes are central to the maintenance of differentiated function in thyroid cancers and loss-of-function mutations in SWI/SNF complexes lead to radioiodine-refractory cancer and resistance to redifferentiation therapies by MAPK pathway inhibitors. If prospectively validated, patients with RAI non-avid lesions with concurrent mutation in SWI/SNF complexes will not be responsive to redifferentiation therapy and will require additional strategies to restore the function of SWI/SNF complexes. In comparison, patients with RAI non-avid lesions carrying *BRAF* or *RAS* mutation without concurrent mutation in SWI/SNF complexes are more likely to benefit from redifferentiation RAI therapy.

Is there a minimum ^131^I absorbed dose required for therapeutic efficacy? What level of RAI delivery enhancement needs to be achieved to improve the ^131^I therapeutic outcome? Lesion dosimetry has been proposed to ensure the delivery of sufficient ^131^I to target lesions. However, lesion-guided dosimetry is cost-prohibitive and rarely employed in clinical practice. Instead, ^131^I radioactivity not exceeding potential bone marrow toxicity or empirical doses is administered to patients with RAI-avid progressive disease, as the superiority of lesion-guided dosimetry on a patient’s clinical outcome has not been proven in randomized controlled trials. One may argue that any level of RAI delivery enhancement to target lesions is beneficial, as it would not only increase the percentage of administered radioactivity to target lesions but also decrease the percentage of administered radioactivity to non-targeted tissues to reduce the risk/severity of adverse side effects. Furthermore, the minimum ^131^I absorbed dose required for therapeutic efficacy would vary among lesions of differential ^131^I radiosensitivity. Accordingly, personalized RAI delivery enhancement should not be limited to patients with RAI non-avid lesions but may also apply to patients who receive ^131^I therapy for known disease. A prospective study should be conducted to investigate whether administration of a BRAF/MEK inhibitor preceding ^131^I therapy results in a better responsiveness to ^131^I therapy and/or lower incidence/severity of adverse effects, especially among patients whose lesions demonstrate an increase in RAI delivery via pretherapeutic RAI scans.

## How do we increase ^131^I radiosensitivity in target lesions?

Differential ^131^I radiosensitivity among various metastatic lesions within an individual as well as varying degrees of radiosensitivity among patients have been clinically recognized but have yet to be systematically investigated. Differential radiosensitivity among thyroid cancer cell lines has been recently reported^5,6^, and thyroid cancer cell lines carrying the *BRAF*^V600E^ mutation appeared to be more radioresistant than cells carrying wild-type *BRAF*^5^. Radiosensitization by administration of a BRAF and/or MEK inhibitor has been reported in melanoma patients^7,8^. Indeed, the BRAF inhibitor vemurafenib has been shown to radiosensitize thyroid cancer cells carrying the *BRAF*^V600E^ mutation by constraining DNA double strand break repair^5^. While the aforementioned studies applied external beam radiation rather than ^131^I, radiosensitivity to external beam radiation will likely closely correlate to radiosensitivity to ^131^I, as both share a common mechanistic action on DNA damage for their therapeutic effects despite their difference in dose rate. Taken together, administration of a BRAF/MEK inhibitor not only increases RAI delivery to target lesions but also may contribute to increasing radiosensitivity to augment ^131^I therapeutic efficacy in thyroid cancer carrying *RAS* or *BRAF* mutations.

As summarized in a recent review 9, resistance to radiation therapy is polymodal and is associated with changes in multiple biological processes such as DNA repair, cellular energetics, growth signaling pathways, inflammation, angiogenesis, and oxygen tension that can occur within the tumor and/or in the surrounding microenvironment. Consequently, therapeutic agents that target these hallmarks of cancer may also enhance radiosensitivity. Of interest, chemotherapy is often used in combination with radiation therapy for anaplastic thyroid cancer because of the radiosensitizing effect of chemotherapeutic agents^10^. ^131^I therapy, a targeted therapy for RAI-avid lesions, has been given as a single therapeutic modality to achieve systemic control for many thyroid cancer patients for the past ~80 years. Given the rapid development of radiosensitizers^11^ and promising clinical outcome of chemoradiotherapy, ^131^I therapeutic efficacy may be further improved by including radiosensitizers that are already clinically approved or in experimental pipelines.

## How does ^131^I cure or stabilize progressive thyroid cancer?

In contrast to external radiation, ^131^I is taken into thyroid cancer cells and is retained inside of cells for various lengths of time depending on the intrinsic iodine retention/organification. The dose rate of radiation for ^131^I is much lower than external radiation but may achieve similar or higher absorbed dose per treatment. Similar to external radiation, it is believed that beta rays emitted from ^131^I decay directly or indirectly cause DNA damage in cancer cells. For cancer cells defective in DNA damage repair, they may not be able to continue cell division passage upon ^131^I-induced DNA damage and may commit to apoptosis. Radiation emitted by ^131^I in RAI-avid lesions can cause damage in surrounding RAI non-avid lesions within 2 mm in distance. Irradiated cells can release signals that reduce the survival of adjacent cells, i.e. bystander or cohort effect^12^. In addition, an abscopal effect contributed by immune surveillance of the exposed neoantigens from dying RAI-avid lesions may eradicate RAI non-avid lesions that share the same neoantigen, which is the same rationale behind local tumor irradiation to enhance the effect of immunotherapy for systemic control. Indeed, external radiation has historically been used to achieve local control yet has recently been proposed to enhance immunotherapy to achieve systemic control for solid tumors^13^. Taken together, the therapeutic efficacy of ^131^I may be impacted by radioactivity delivered to target lesions and differential radiosensitivity among lesions as well as the presence or absence of bystander and/or abscopal effect.

**Figure 1.**
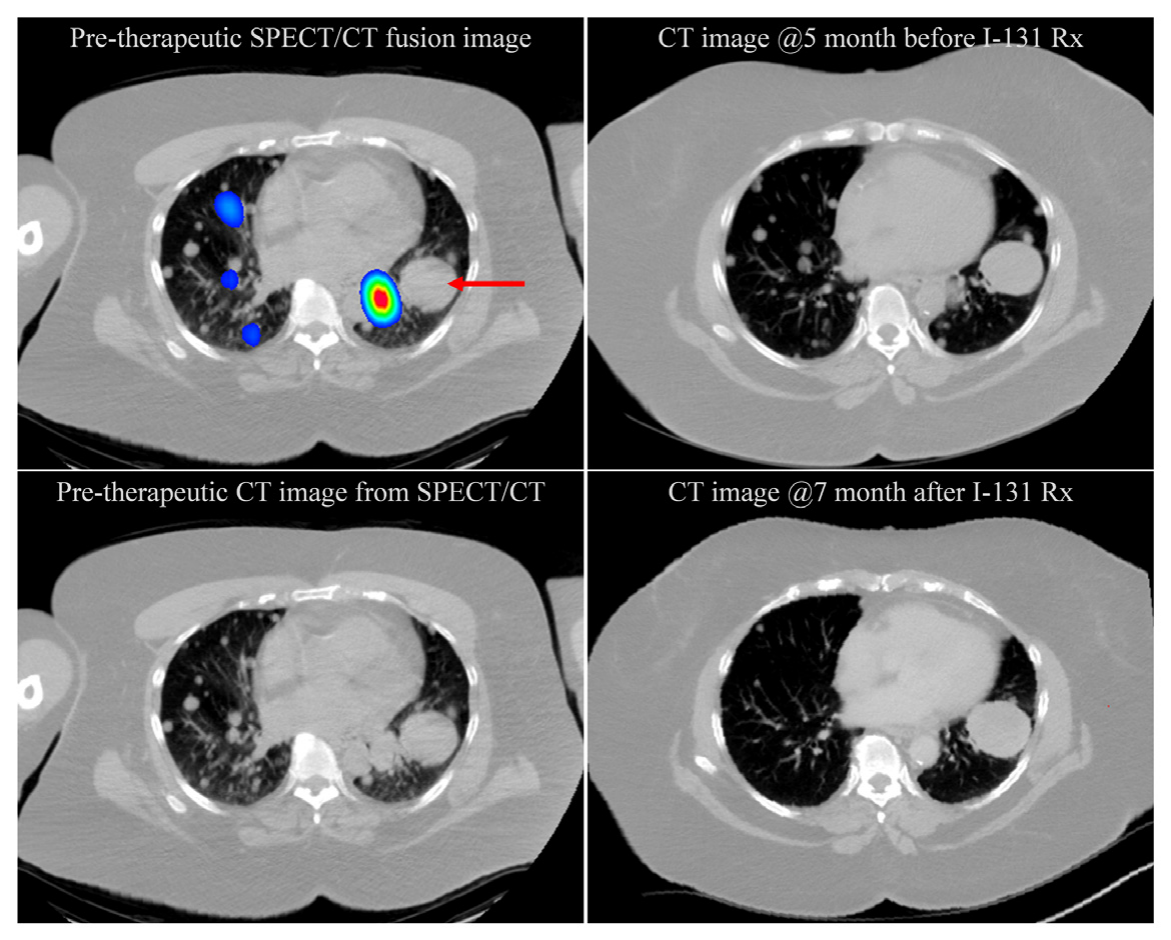
Pretherapeutic ^123^I SPECT/CT image and CT chest images prior to and after ^131^I therapy. Pretherapeutic ^123^I SPECT/CT fusion image shows heterogeneity in radioactive iodine (RAI) avidity among metastatic lung lesions, with the largest lesion being RAI non-avid (red arrow). Seven months after ^131^I treatment (121 mCi), ~160 lesions that include RAI-avid and RAI non-avid lesions were no longer detectable.

Patients with RAI non-avid index lung lesion(s) in the presence of concurrent RAI-avid lesions are often categorized as RAI refractory. These patients generally do not benefit from ^131^I therapy. Here, we show a patient who was super responsive to ^131^I therapy most likely due to the combination of the lesion’s radiosensitivity with the presence of both bystander and abscopal effects. As illustrated in Figure 1, a pretherapeutic ^123^I SPECT/CT fusion image shows heterogeneity in RAI avidity among metastatic lung lesions, with the largest lesion being RAI non-avid. After 121 mCi of ^131^I treatment, numerous lesions that include RAI-avid and RAI non-avid lesions were no longer detectable in the follow-up CT chest images. As determined by our in-house software, the largest RAI-avid and RAI non-avid lung lesion that disappeared at 7 months after ^131^I treatment had a lesion volume of 29,398 mm^3^ and 1,804 mm^3^ prior to ^131^I therapy, respectively. Taken together, the patient’s responsiveness to ^131^I therapy appears to be multifactorial and goes beyond lesion dosimetry.

## How do we achieve personalized radioiodine therapy for patients with known disease after thyroidectomy?

For patients with known disease, complete response to ^131^I therapy is currently limited to a few patients with RAI-avid micro-metastases. Partial responses or stable disease can be accomplished by ^131^I therapy in many patients, yet a few patients with RAI-avid progressive disease do not respond to ^131^I therapy. Furthermore, patients with RAI non-avid lesions likely would not benefit from ^131^I therapy if their lesions do not respond to a re-differentiation regimen prior to ^131^I therapy. With more re-differentiation agents and radiosensitizers becoming clinically available, as well as the option of a combination with immunotherapy, we anticipate many more thyroid cancer patients will benefit from genetic variant-guided and/or data-driven personalized ^131^I therapy. The challenges and opportunities ahead are identifying patients who “need” and “could benefit from” enhancement of RAI delivery, RAI radiosensitivity, and/or immune competence. In addition, the duration and type of regimen of the aforementioned enhancements need to be optimized for individual patients.

Currently, pretherapeutic ^123^I-SPECT or ^124^I-PET imaging helps us to identify patients in need of RAI delivery enhancement and to evaluate the efficacy of the intervention. A recent paper reported that lesion uptake of both ^18^F-FDG (fluorodeoxyglucose, a marker of glycolytic activity) and ^18^F-FAZA (fluoroazomycin arabinoside, a marker of hypoxia) is associated with short-term progression after ^131^I therapy^14^. Pretherapeutic PET imaging with ^18^F-FAZA or ^18^F-FMISO (fluoromisonidazole, a marker of tissue hypoxia) allows us to identify patients in need of a radiosensitizer targeting tumor hypoxia and to evaluate the efficacy of the intervention. While imaging technologies that monitor the dynamics of the immune system are still under development, multiple imaging modalities and immunologic targets are being actively explored^15^. Anatomic imaging allows us to monitor changes in lesion volume and lesion volume doubling time such that the speed of disease progression and the extent of responsiveness to ongoing therapeutic intervention can be closely monitored to provide guidance on timely clinical intervention.

In the future, with the rapid growth of big data analytics in genomics, imaging, and clinical outcomes, it would be ideal to generate a personalized, data-driven risk profile to customize ^131^I therapy for individual patients aiming for both long-term tumor control and manageable adverse effects. For example, specific germline variants reflecting tumor microenvironment hypoxia and/or compromised immune function, along with somatic tumor mutations reflecting DNA repair deficiency, may serve as genomic signatures for RAI radiosensitivity. Moreover, the predictive power of a data-driven machine learning radiogenomic model has been shown to be superior to dosimetric- or genomic-only mechanistic modeling strategies in radiation oncology^16^. Applying deep learning algorithms to big data composed of radiogenomics, demographic and histopathological data, and clinical outcomes may extract data signatures indicative of RAI avidity, RAI radiosensitivity, and immune competence that are required to achieve best ^131^I therapeutic efficacy. Consequently, personalized data-driven risk profiles predictive of deficiency in RAI radiosensitivity, RAI delivery, and/or immune competence may be identified and may also provide tailored strategies to overcome the deficiency. Taken together, personalized ^131^I therapy for thyroid cancer patients with known disease will become a reality in the near future.
